# Integrated effects of top-down attention and statistical learning during visual search: An EEG study

**DOI:** 10.3758/s13414-023-02728-y

**Published:** 2023-06-01

**Authors:** Carola Dolci, C. Nico Boehler, Elisa Santandrea, Anneleen Dewulf, Suliann Ben-Hamed, Emiliano Macaluso, Leonardo Chelazzi, Einat Rashal

**Affiliations:** 1 Department of Neuroscience, Biomedicine, and Movement Science, University of Verona, Strada le Grazie, 8, 37134 Verona, Italy; 2 Department of Experimental Psychology, Ghent University, Ghent, Belgium; 3 Institut des Sciences Cognitives Marc-Jeannerod, Lyon, France; 4 Lyon Neuroscience Research Center, Lyon, France

**Keywords:** N2pc, P1, Statistical learning, Endogenous cueing, Attention control, Priority map

## Abstract

The present study aims to investigate how the competition between visual elements is solved by top-down and/or statistical learning (SL) attentional control (AC) mechanisms when active together. We hypothesized that the “winner” element that will undergo further processing is selected either by one AC mechanism that prevails over the other, or by the joint activity of both mechanisms. To test these hypotheses, we conducted a visual search experiment that combined an endogenous cueing protocol (valid vs. neutral cue) and an imbalance of target frequency distribution across locations (high- vs. low-frequency location). The unique and combined effects of top-down control and SL mechanisms were measured on behaviour and amplitudes of three evoked-response potential (ERP) components (i.e., N2pc, P1, CNV) related to attentional processing. Our behavioural results showed better performance for validly cued targets and for targets in the high-frequency location. The two factors were found to interact, so that SL effects emerged only in the absence of top-down guidance. Whereas the CNV and P1 only displayed a main effect of cueing, for the N2pc we observed an interaction between cueing and SL, revealing a cueing effect for targets in the low-frequency condition, but not in the high-frequency condition. Thus, our data support the view that top-down control and SL work in a conjoint, integrated manner during target selection. In particular, SL mechanisms are reduced or even absent when a fully reliable top-down guidance of attention is at play.

## Introduction

In everyday life, the large number of visual inputs coming from the environment greatly exceeds our sensory and cognitive processing capacities. Looking for a book in a crowded library can be a difficult task since, at all times, all the available visual stimuli compete with each other in order to gain access to further processing. Visual attention is the cognitive function that acts as a filter, allowing us to select the relevant information and tune out what is irrelevant (Desimone & Duncan, 1995; Reynolds & Chelazzi, 2004). This process involves one or multiple attentional control (AC) mechanisms that assign attentional priority to a certain stimulus or location in the visual field. A prominent theory of attentional guidance is the *priority map* theory, which suggests a neural representation of visual space that is topographically organized (Bisley & Goldberg, 2010; Ptak, 2012). Depending on the context and time, each location in the visual space is suggested to have a specific level of neuronal activity that is determined by the amount of attentional priority assigned to that location (Awh et al., 2012; Chelazzi et al., 2013; Di Bello et al., 2022; Ipata et al., 2009; Serences & Yantis, 2007). The highest activation peak triggers a winner-takes-all process, leading to the target at that location being selected (Bisley, 2011; Chelazzi et al., 2014; Macaluso & Doricchi, 2013; Noudoost et al., 2010). Thus, the distribution of attentional resources within the priority map would be influenced by the action of different AC mechanisms.

Individual priority signals may originate from various sources. Traditionally, they have been separated into two main categories: *top-down* and *bottom-up*. Top-down (or goal-directed) AC is an endogenous process, driven by active volitional selection of items that are relevant to a person’s goals or instructions (Carrasco, 2011; Egeth & Yantis, 1997; Leber & Egeth, 2006; Parisi et al., 2020; Reynolds & Heeger, 2009). For instance, the competition between stimuli can be solved by the presence of a central visual cue, which indicates the forthcoming target location and allows the pre-allocation of attentional resources to that position, facilitating target detection (Posner, 1980). In contrast, bottom-up attention is an exogenous AC mechanism by which attentional resources are automatically allocated toward a salient stimulus with highly noticeable feature properties, such as luminance, color, or shape (Theeuwes, 1991, 1994; Theeuwes & Godijn, 2004; Yantis & Egeth, 1999).

In recent years, it has been shown that people can implicitly develop another type of bias specifically linked to the individual’s previous experience with a given context and/or stimuli, which can also guide target selection (Awh et al., 2012; Ferrante et al., 2018; Jiang, 2018). Therefore, a third AC category has been introduced: *experience-dependent* AC (Awh et al., 2012; Chelazzi & Santandrea, 2018; Failing & Theeuwes, 2018). One of the experience-dependent mechanisms is *statistical learning* (SL), which allows humans, but also other animals, to implicitly extract regularities from the environment even without having explicit instructions (Aslin & Newport, 2012; Druker & Anderson, 2010; Ferrante et al., 2018; Duncan & Theeuwes, 2020; for evidence in non-human primates: Newport et al., 2004; and chicken: Rosa-Salva et al., 2018; Santolin et al., 2016). In particular, the probability with which a target element occurs in a specific location was found to induce an attentional bias toward the location where it is more likely to appear, without the participant being consciously aware of this (Geng & Behrmann, 2002, 2005).

As described above, many studies investigated how visual attention is guided by individual AC mechanisms, but they do not specify how attentional selection is reconfigured when different attentional biases are at a play. Indeed, in many aspects of everyday life multiple AC mechanisms can act simultaneously, and it is still unclear how they interact with one another in the prioritization process and how the final attentional choice is established. One possibility is that, when acting simultaneously, the activity of all the AC mechanisms is added-up and they jointly contribute to solving the competition in favour of one stimulus. Alternatively, one mechanism may prevail over the other, thus exclusively governing target selection.

The aim of the present study was to study the combination of AC mechanisms, specifically, top-down AC and statistical learning, and to test the hypotheses of a joint contribution versus mechanism prevalence in solving the competition between stimuli. Previous studies are more in line with the first hypothesis, arguing that these two mechanisms operate independently from each other, with the influence of the two adding up in a linear manner when engaged at the same time (Duncan & Theeuwes, 2020; Gao & Theeuwes, 2020; Geng & Behrmann, 2005). For instance, Gao and Theeuwes (2020) showed how SL biased the competition in favour of a target that appeared frequently in a ceratian location, compared with a target that appeared in a rare location in the array, and that this effect was not affected by top-down attention being directed to one or the other location. At the same time, better performance was found when participants could benefit from valid (vs. invalid) information that was given to the top-down AC mechanism, when the target was presented in both the high- and the low-frequency locations (Gao & Theeuwes, 2020). This suggests that both mechanisms can guide independently the attentional selection of specific spatial locations on the priority map.

However, in that study, top-down control was always present, as participants were instructed to attend to a location in the array that could correspond to the location of the upcoming target, but not with complete certainty; only on 50% of the trials the cue indicated the exact target location, whereas on the other half of the trials it indicated a location nearby. Thus, it is possible that the cue validity led to the absence of an observable interaction between the two mechanisms, as the level of uncertainty introduced in the paradigm may have prevented a possible interaction between the mechanisms. For this reason, in the current study we provided participants with a fully predictive top-down control guidance, by using a 100% valid cue that pointed to the upcoming target location and compared it to a condition where top-down control was not at a play, where participants were provided with an uninformative neutral cue.

### EEG markers of visual selective attention

To further examine the prioritization process in visual search, in the present study, we investigated the neural mechanism underlying it. Specifically, we focused on three well-established evoked-response potential (ERP) components related to attentional selection: the cue-related contingent negative variation (CNV), and the target-related P1 and N2pc. When investigating selective attention using visual-search paradigms, the main EEG marker of interest is the N2pc, which is the negative deflection at posterior electrodes contralateral to the target, typically emerging around 200 ms from the onset of a lateralized target. Traditionally, the N2pc has been assumed to index the shift of covert attention toward a task-relevant, or salient, stimulus (Eimer, 1996; Luck & Hillyard, 1994), but other findings suggest that the N2pc reflects various aspect of target processing (Kiss et al., 2008; Theeuwes, 2010; Zivony et al., 2018). Relevant for the current study, in a recent work using a very similar task to the one used here, Rashal and colleagues (2022; Experiment 2) observed an N2pc for targets preceded by a valid endogenous cue to the target location, suggesting that the N2pc reflected attentional processes also following topdown deployment of attention to that location.

As SL induces a change of attentional priority in favour of the high-frequency target location, it might be expected that a facilitation of target selection (Ferrante et al., 2018; Geng & Behrmann, 2002) would be accompanied by a larger N2pc elicited by that target. Still, a recent study by van Moorselaar and Slagter (2019) found instead a reduction of N2pc amplitudes when the target appeared frequently in a certain location. In their study, however, the target competed with only one other stimulus (i.e., the distractor), making the task easier as attentional selection was quickly accomplished (see also Rashal et al., 2022, for evidence that the N2pc is modulated by difficulty-related factors). Furthermore, statistical learning in that study was constrained to target repetitions, with the target appearing at the same location for a number of consecutive trials (4 trials) within a sequence, but that location varied across the duration of the experiment. Critically, the modulation of the N2pc reported by van Moorselaar and Slagter (2019) revealed that the N2pc amplitude, and thus the deployment of attentional resources needed for target selection, was diminished for repeating target location in consecutive trials. That is, the N2pc was larger in the first trial than in the last trial of the repetition sequence. However, this result may be attributed to inter-trial priming and may not apply to a situation where SL is established across the entire experiment. As a matter of fact, in classic SL paradigms, target location frequency is associated with just one (or a few) spatial location(s) or region(s) across the entire experiment, allowing SL to be reinforced continuously and inducing an attentional enhancement in favour of that location.

Two other components related to attentional control are the post-cue CNV and the post-target P1 (Mangun, 1995; Schevernels et al., 2014; Van Den Berg et al., 2014), the first specifically related to top-down control, while the latter is potentially modulated by top-down and bottom-up mechanisms. The CNV is characterized by a slow, negative-going waveform normally detected in central areas after the presentation of a warning stimulus such as a cue (Walter et al., 1964), likely reflecting a general preparatory attention during the cue-target interval of attentional tasks (e.g., Grent-‘t-Jong & Woldorff, 2007). The P1 is the first positive-going ERP component, starting around 90 ms after target-array onset, and displays increased amplitudes over the occipital scalp contralateral to the precued location (Baumgartner et al., 2018; Mangun & Hillyard, 1991). P1 amplitudes have been demonstrated to be enhanced when the corresponding visual stimuli appeared on the cued compared with the non-cued side of the array, suggesting that it is an early manifestation of top-down attentional control (Eimer, 1994; Mangun & Hillyard, 1991; Van Voorhis & Hillyard, 1977).

### Aim and hypotheses of the study

We devised a visual search task to investigate both isolated and integrated effects of different sources of AC during the target selection process. In particular, we focused on top-down attention control, which we manipulated via endogenous cueing, and statistical learning, which was manipulated by an imbalance of target frequency across locations. By comparing performance in trials where targets appeared in the *high*- (HFTL) versus *low*- frequency target location (LFTL) and were preceded by an informative (valid) or non-informative (neutral) cue, we tested whether top-down control and SL, when active together, both contribute to assigning attentional priority to a specific spatial location (hypothesis 1) or if one mechanism is blocked by the presence of the other mechanism (hypothesis 2). Specifically, if the two mechanisms operate independently, better performance and a larger N2pc should be observed for targets in the HFTL compared with the LFTL condition irrespective of the cueing condition. At the same time, cueing effects should emerge regardless of the target location frequency condition, and better performance and a larger N2pc should emerge following a valid cue when the target appears in both the HFTL and the LFTL (for behavioural evidence, see Gao & Theeuwes, 2020). Alternatively, if the two mechanisms interact with each other, we should find that one mechanism affects the other in some way. For example, it is possible that top-down control blocks the effect of SL, such that its effect can be reduced or even gated by pre-cueing the target location, resulting in a smaller difference in performance and N2pc mean amplitude between targets in the HFTL and LFTL following a valid cue compared with the same difference in performance and N2pc amplitudes for targets following a neutral cue. Similarly, it can be that SL blocks top-down control. In this case, we should find that the benefit of validly cueing the target location is reduced by the presence of target-location frequency imbalance, resulting in a smaller cueing effect on behaviour and N2pc amplitude in the HFTL compared with the LFTL conditions.

Additionally, we examined two other EEG components mostly related to the top-down control: the P1 during visual search, and the CNV during the cue-target interval. By looking at the CNV and P1 components, we can investigate modulations to top-down attentional orienting pre- and post-stimulus array onset. A larger CNV should emerge following a valid compared with a neutral cue, reflecting advance preparation for selecting the target stimulus (Rashal et al., 2022; Schevernels et al., 2014; Van Den Berg et al., 2014). Furthermore, the P1 could also be modulated by the presence or absence of a valid cue. Specifically, we expected a larger P1 following a valid compared with a neutral cue, indicating an early stimulus categorization when a stimulus is presented in the expected spatial location (Heinze et al., 1994; Mangun, 1995). Indeed, Livingstone and colleagues (2017) demonstrated that P1 indexes an enhanced processing for the search item pointed by a valid cue at a stage of vision that precedes attentional selection.

Lastly, even if a modulation of the CNV and P1 have been most clearly associated with cueing, here we tested if SL was able to affect the general preparation and attentional orienting pre- and post-stimulus array onset. If so, as for N2pc, we would expect a larger CNV and P1 for targets in the HFTL, compared with the LFTL condition, and this effect could interact with the cueing manipulation.

## Methods

### Participants

Twenty-four healthy volunteers (four males; mean age 23.62 years, SD ±3.4 years) with normal or corrected-to-normal visual acuity participated in this experiment. None of them had previously taken part in similar or related studies, and they were naive to the purpose of the present study. At the end of the experiment, they received a fixed monetary compensation for their participation (€32.5). All subjects gave their written informed consent before participation. The present study was approved by the ethics committee of the Faculty of Psychology and Educational Sciences of Ghent University (code 2021/09).

### Apparatus and stimuli

The experiment was conducted in a dimly lit and quiet room, where participants sat in front of a 24-in. Benq XL2411Z LED monitor controlled by a Dell Optiplex 9020 tower with Intel Core i5-4590 processor, at 60-Hz refresh-rate. The viewing distance was held constant at 60 cm by using an adjustable chin rest. The experiment was run with the PsychoPy (v1.84.2) software (Peirce, 2007). Good central fixation by the participants was monitored using the camera of an Eyelink 1000 plus (SR Research, Canada). The experimenter was sitting in a different room and warned the participants during breaks in case eye-movements were observed in the preceding block, to allow correction.

**Fig. 1:**
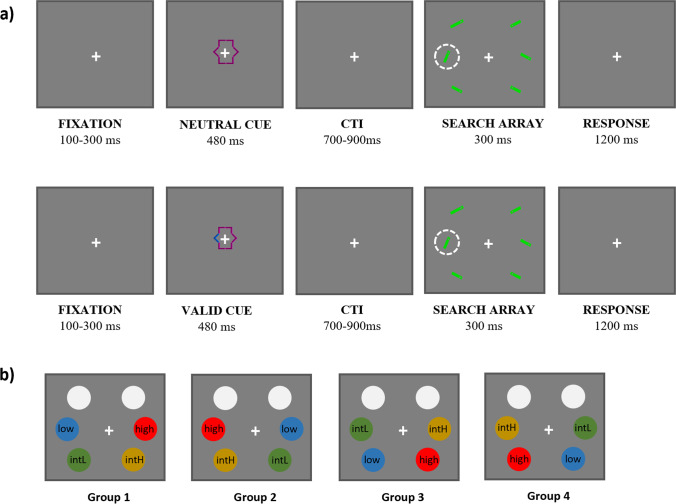
**a** Examples of the trial sequence. Top row: a neutral cue preceded target array onset. Bottom row: target location was predicted by a valid cue. The target is indicated in the figure by the dashed circle (for illustration purposes; no such circle was present during the task) and was the bar tilted ±25° from its vertical axis, while the non-targets were tilted ±25° from their horizontal axis. **b** Target frequency distribution across groups (during neutral cue trials only). Note that the target never appeared in the two upper locations

The stimuli were rectangular bars of 2.0° × 0.5° in size, either green (RGB coordinates: 0, 86, 0; luminance: 138.5 cd/m^2^) or red (RGB values: 170, 0, 0; luminance: 64.8 cd/m^2^), presented on a grey background (RGB: 128, 128, 128; luminance: 85.5 cd/m^2^). Colours were randomly chosen on a given trial, with all stimuli being drawn in the same colour. The choice for two colours, which was not essential to the present task, and which was fully counterbalanced across conditions, largely relates to earlier work of ours using the same global approach (Rashal et al., 2022). Within each stimulus, there was a small gap (diameter of 0.25°) of the same grey colour as the background and positioned at the upper or lower part. The target was a bar tilted ±25° across the vertical axis, whereas the other stimuli that had to be ignored (distractors) were bars tilted ±25° across the horizontal axis. Two stimuli were presented in the upper visual field, two on the horizontal midline and two in the lower visual field (Fig. 1, panel a). Since evidence indicates that the N2pc is usually larger in the lower visual field and on the horizontal meridian than in the upper visual field (Bacigalupo & Luck, 2019; Luck et al., 1997), the target never appeared in the two upper locations, which hence just contained filler items. In each visual search display, six stimuli were presented, centred equidistantly 7° away from a white fixation cross (0.5°×0.5°; RGB: 255, 255, 255; luminance 190.2 cd/m^2^).

Before the onset of the stimulus array, a cue stimulus was presented around the fixation cross (Fig. 1, panel a). The cue consisted of a geometric shape (dimension: 1.5° × 1.5°) made up of six separate corners, each pointing at one of the stimulus locations. In the case of the neutral cue, all the corners were coloured with the same pink (RGB: 120, 0, 90; luminance: 89.5 cd/m^2^), whereas in the case of the valid cue, five corners were pink and one was cyan blue (RGB: 0, 56, 158; luminance: 81.1 cd/m^2^), indicating in which spatial position the target element would be presented (Fig. 1, panel a).

### Experimental design

A central cue presented prior to the target array onset was either valid or neutral. In the valid cue condition, the location of the upcoming target was predicted with a validity of 100%. In the neutral cue condition, the cue did not include information about the target location. Importantly, in order to not mix the two AC manipulations, SL was manipulated exclusively following neutral cues by introducing, unbeknown to the participants, an imbalance of target frequency appearance across the four possible target locations: high, low and two intermediate location frequencies (Ferrante et al., 2018). The valid cue, when present, indicated with equal frequency each of the four possible target locations (96 trials each location). The neutral cue trials (1,216 trials; 76% of all trials) provided a baseline where the SL effect could be assessed in the absence of top-down guidance. Here the target appeared in the high-frequency location 50% of the trials (608 trials), in the low-frequency location for 7.9% of the trials (96 trials), and in each of the intermediate-frequency locations for 21% of these trials (256 trials each). We did not introduce an imbalance of target frequency appearance in the valid cue condition because doing so would mean introducing an imbalance of valid cues. This, in turn, would complicate the interpretation of the results, as it would be impossible to disentangle the benefit in target detection due to SL of the target location, or SL of the valid cue, or both. Participants were randomly assigned to one of four groups (Fig. 1, panel b), each with a different spatial configuration of target-location probabilities, but with the constraint that the high-probability and low-probability conditions were always in opposite locations in the left versus right visual field.

### Procedure

Each experimental trial (Fig. 1, panel a) started with a fixation cross, which remained on the screen for the whole duration of the experiment. After a random interval jittered between 100 and 300 ms, the cue appeared for 480 ms. After a cue-target interval (CTI), jittered between 700 and 900 ms, the search display appeared and remained visible for 300 ms. Responses were recorded from the onset of the search display until 1,200 ms after display offset, for a total of 1,500 ms. Afterwards, a new trial sequence started automatically. The task was to discriminate the position of the gap within the target item (top vs. bottom) by pressing the letter ‘M’ on the keyboard with their right index finger if the gap was in the lower part, or the letter ‘Z’ with their left index finger if it was in the upper part. The experiment included a total of 1,600 trials, divided into eight blocks. Before starting the actual experiment, a practice phase of 64 trials was used to allow participants to familiarise themselves with the task. All the conditions previously described were presented in a fully randomized order. Participants were instructed to maintain their eyes on the fixation cross, and fixation quality was monitored by the experimenter by means of the online eye-position display of the eye-tracker.

In order to evaluate if participants were aware of the frequency manipulation, a survey was conducted at the end of the experiment (see Ferrante et al., 2018). Participants were first asked to report whether they noticed something about the spatial distribution of target stimuli, and in case they responded “yes”, they had to report (or guess) the locations where the target was presented most frequently.

### Electrophysiological recording and analysis

EEG data were recorded using a Brain Products actiCHamp 64-channel system (Brain Products, Gilching, Germany) with 64 active scalp electrodes positioned according to the standard international 10–10 system. Signals were recorded at a 500-Hz sampling rate, using Fz as the online reference and then re-referenced offline to the average of TP9 and TP10, corresponding to the left and right mastoids. Fz was then restored to the dataset. A high-pass filter of 0.1 Hz was applied to the raw data and segments of the continuous data, with clearly identifiable, large artefacts (not including blinks and eye movements) were excluded by manual inspection. Successively, independent component analysis (ICA) was used to remove components related to eye blinks and (residual) eye movements. We then segmented the data into epochs from −200 ms to 2,900 ms relative to cue onset and from −200 ms to 800 ms relative to the stimuli array onset. We then baseline-corrected with regard to the 200-ms pre-cue or pre-stimuli period, respectively. Then, a second artifact rejection (AR) was performed to flag and remove epochs that contained artefacts in the analysed channels (PO7/8; absolute amplitude exceeding ±100 μV). On average this led to exclusion less than 3% of the total trials.

In order to study the temporal dynamics of attentional orienting and subsequent visual search, we focused on three components, namely the cue-evoked CNV and the P1 and N2pc elicited by the search array.^1^ The CNV was examined at Cz using the cue-locked epochs (Verleger et al., 1999; Rashal et al., 2022), whereas for the N2pc and P1 we used the average of two electrodes capturing activity at PO7/PO8, where the N2pc and P1 are usually the largest (e.g., Liesefeld et al., 2017; Rashal et al., 2022). To determine the analysis time-windows for each of these EEG markers, we took the canonical values used in the literature: for the CNV, we selected a time-range from 700 ms after cue onset until approximately the earliest point in the CTI in which the search display could appear (plus 70 ms, accounting for transduction delay into visual cortex), i.e., 700–1,250 ms (e.g., Liebrand et al., 2017; Rashal et al., 2022). For the N2pc and P1, the respective time-ranges were set to 200–300 ms (N2pc) and 90–140 ms (P1) after the search-display onset, in line with the existing literature (e.g., Eimer, 1996; Luck et al., 2000; Mangun & Hillyard, 1991). Note that counter to most of the earlier N2pc and P1 literature, the target location frequency imbalance led to the fact that for a given participant, the contralateral and ipsilateral locations were either PO7 or PO8, and could not be collapsed across those locations for different conditions, with corresponding targets on the left and right (e.g., Wu et al., 2011). Therefore, in this study, the average across locations was possible only across groups (Wang et al., 2019).

Analyses were performed using R 3.6.2 (R Core Team, 2016) with ez (Lawrence, 2011/2015) and effectsize (Ben-Shachar et al., 2020) packages. For CNV we used rm-ANOVAs to compare the mean amplitude in the different conditions, whereas for N2pc and P1 we first calculated the mean amplitude of the ipsi and contra location of interest, and then performed rm-ANOVAs to compare the difference waves (DWs) resulting from the subtraction *contra-minus-ipsi* between different conditions. All these analyses were performed only using trials with correct responses. P values were corrected with Greenhouse-Geisser epsilon in cases of significant sphericity violation.

## Results

### Behaviour

**Fig. 2:**
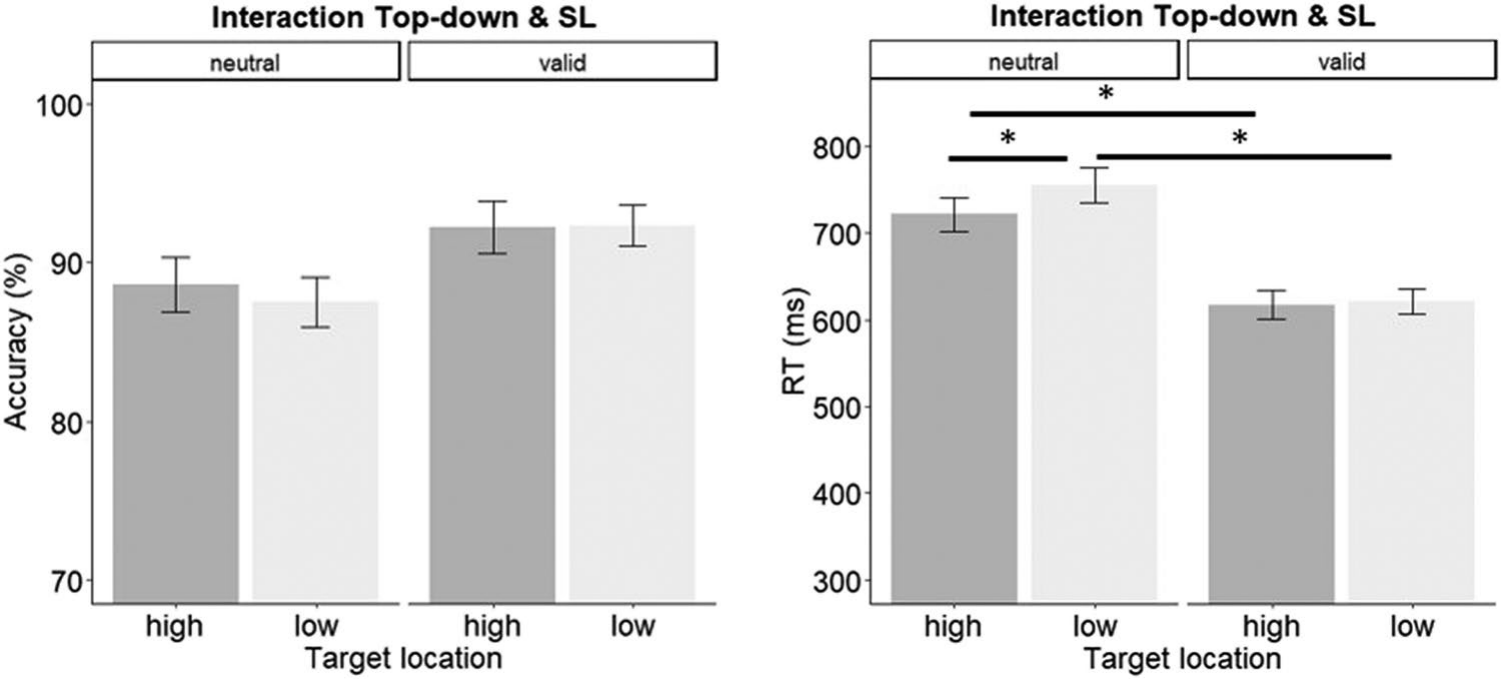
Mean accuracies (**left**) and reaction times (RTs; **right**) as a function of cue and target frequency conditions. Error bars represent the standard error of the mean

In order to assess the effects of and interaction between statistical learning and top-down mechanisms, 2 × 2 rm-ANOVAs were conducted with Target Location Frequency (high, low) and Cue (valid, neutral) for accuracy and reaction time (RT). These analyses showed significant main effects of Cue for accuracy and RT [ACC: *F*(1, 23) = 15.32, *p* = 0.0006, η^2^_p_ = 0.39; RT: *F*(1, 23) = 134.41, *p* < 0.0001, η^2^_p_ = 0.85], and Target Location Frequency for RT [ACC: *F*(1, 23) = 0.46, *p* = 0.50, η^2^_p_ = 0.01; RT: *F*(1, 23) = 6.10, *p* = 0.02, η^2^_p_ = 0.20]. Importantly, a significant interaction between the two factors was observed for RT [ACC: *F*(1, 23) = 0.71, *p* = 0.40, η^2^_p_ = 0.03; RT: *F*(1, 23) = 9.32, *p* = 0.005, η^2^_p_ = 0.29]. Post hoc paired t-tests (two-tailed) revealed that participants were significantly faster in detecting the target in the HFTL compared with the LFTL, but only when the cue was neutral [*t*(23) = -2.84; *p* = 0.009, Cohen’s *d =* -0.34, -30 ms], and not when the cue was valid [*t*(23) = -0.73; *p* = 0.47, Cohen’s *d =* -0.04, -3 ms], suggesting that top-down control is able to exert a gating effect over SL mechanisms.^2^ Furthermore, the benefit of the valid cue was observed in both the HFTL [*t*(23) = 12.25; *p* < 0.0001, Cohen’s *d =* 1.02, 100 ms] and the LFTL [*t*(23) = 9.75; *p* < 0.0001, Cohen’s *d =* 1.49, 130 ms] conditions (Fig. 2), but with a larger benefit for the cue in the latter (130 vs. 100 ms).

At the end of the experimental session, four participants reported having noticed something peculiar regarding the target frequency, and identified the correct high-frequency spatial location as the location where the target was more likely to appear. However, excluding these participants from the analysis did not change the main results, corroborating the implicit nature of the learning process.^3^

### N2pc

To investigate the effect of top-down AC and SL on the N2pc, we first investigated if the component was present in each condition. One-sample t-tests (one-tailed) showed a marginally significant N2pc (mean amplitude lesser than zero) for targets at the HFTL (following neutral cue: t(23) = −1.59, p = 0.06, Cohen's *d =* −0.32; following valid cue: t(23) = −1.57, p = 0.06, Cohen's *d =* −0.32), but not for targets at the LFTL (following neutral cue: t(23) = 0.25, p = 0.59, Cohen's *d =* 0.05; following valid cue: t(23) = −0.90, p = 0.18, Cohen's *d =* −0.18).

**Fig. 3:**
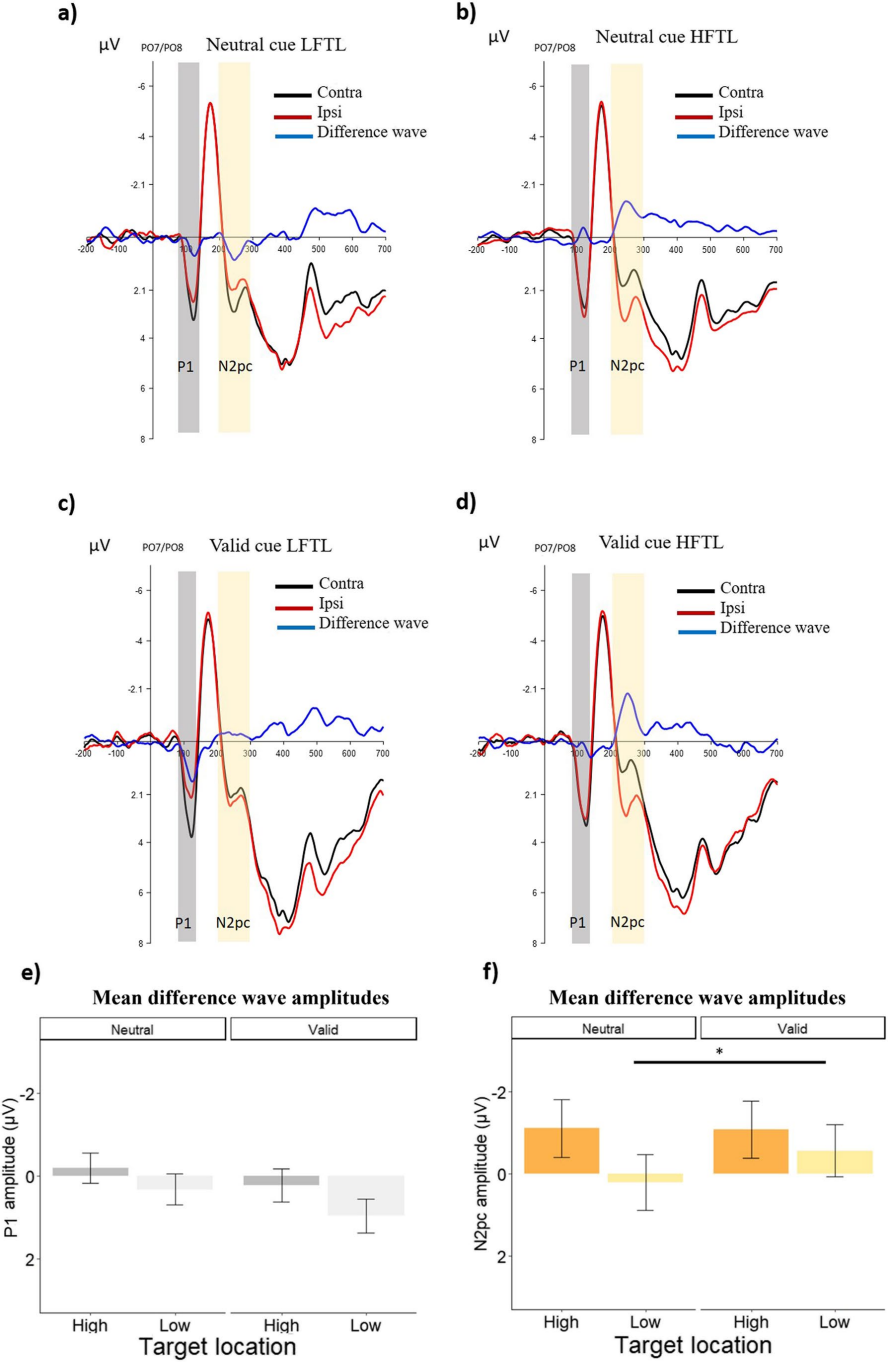
Sensor plots showing contra (black line), ipsi (red line) and the difference waves (contra-minus-ipsi; blue line) activity following a neutral cue (**a**, **b**), or a valid cue (**c**, **d**). Panels a and c depict activity in the LFTL condition, and panels b and d depict activity in the HFTL condition. Time-point zero indicates the search-display onset. The grey area is the time-window where mean amplitude of the P1 was calculated, whereas the yellow area refers to the N2pc time-range. Panels e and f show the mean amplitude of P1 **(e)** and N2pc **(f)**, calculated by subtracting the contra-minus-ipsi channel, in the two Target Location Frequency conditions as a function of the cue. Error bars in plots e and f represent the standard errors of the mean

An rm-ANOVA was then conducted with Cue (valid, neutral) and Target Location Frequency (high, low). This analysis considered the contra-minus-ipsi difference waves, directly focusing on attentional lateralization effects. This analysis showed a significant main effect of Cue [*F*(1,23) = 5.84, *p* = 0.023, η^2^_p_ = 0.20], but not of Target Location Frequency [*F*(1,23) = 0.47, *p* = 0.497, η^2^_p_ = 0.02]. Importantly, a significant interaction emerged between the two factors [*F*(1,23) = 4.28, *p* = 0.049, η^2^_p_ = 0.15]. Post hoc paired t-tests (two-tailed) revealed that targets in the LFTL condition following a valid cue elicited a larger N2pc compared with targets at that location following a neutral cue [*t*(23) = 2.85, *p* = 0.009, Cohen’s *d =* 0.22; 0.74 μV]. In contrast, this effect was not present for targets in the HFTL condition [t(23) = -0.10, *p* = 0.918, Cohen’s *d =* -0.006; -0.02 μV]. Furthermore, no significant difference in N2pc amplitudes was found between HFTL and LFTL, either in the neutral [*t*(23) = -0.94, *p* = 0.355, Cohen’s *d =* -0.37; -1.28 μV] or in the valid cue condition [*t*(23) = -0.40, *p* = 0.688, Cohen’s *d =* -0.16; *-*0.51 μV] (Fig. 3).

### CNV

**Fig. 4:**
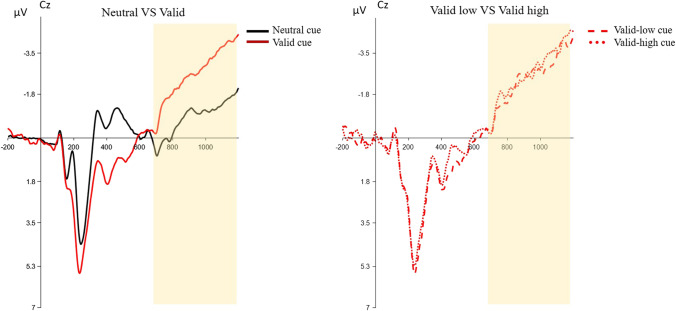
The plot on the left shows the CNV (contingent negative variation) elicited by neutral (black line) and valid (red line) cues, whereas the plot on the right shows the CNV elicited by the valid cue pointing to the LFTL (dashed line) and HFTL (dotted line). Time-point zero indicates cue onset. The yellow area represents the time-window where the mean amplitude of the CNV was quantified

To assess whether a valid endogenous cue elicited a preparatory effect, a one-way rm-ANOVA was performed with Cue (valid, neutral) on the CNV component. This analysis showed a significant difference between the two conditions [*F*(1,23) = 33.46, *p* < 0.001, η^2^_p_ = 0.59]. Specifically, a larger CNV was evoked by valid cues, indicating that the participants could prepare to orient their attentional resources before the search array onset following an informative cue. Furthermore, we explored whether SL proactively modulates top-down control, such that a preparatory effect would emerge according to the target location frequencies. To this end, another rm-ANOVA was conducted on the data from trials following a valid cue in the HFTL and LFTL conditions. No significant difference was found between these two conditions [*F*(1,23) = 0.70, *p* = 0.41, η^2^_p_ = 0.02] (Fig. 4).

### P1

Similar to the analysis conducted for the N2pc, we performed a one-sample t-test (one-tailed) to test whether P1 was meaningfully lateralized in each condition (mean amplitude greater than zero). Results showed a significant lateralization for targets in the LFTL following a valid cue (t(23) = 2.30, p = 0.01, Cohen's *d =* 0.46), but not following a neutral cue (t(23) = 0.76, p = 0.22, Cohen's *d =* 0.15), and not for targets in HFTL (following neutral cue: t(23) = −0.53, p = 0.70, Cohen's *d =* −0.10; following valid cue: t(23) = 0.55, p = 0.29, Cohen's *d =* 0.11).

A two-way rm-ANOVA with Cue (valid, neutral) and Target Location Frequency (high, low) was performed to investigate the effects of top-down AC and SL on the early stage of target selection. Importantly, this was done on the contra-minus-ipsi difference waves, hence characterizing lateralization effects. Similar to the CNV, this analysis revealed a significant main effect of Cue [*F*(1,23) = 23.20, *p* < 0.001, η^2^_p_ = 0.50] that elicited a larger P1 lateralization for valid (vs. neutral) cues, but not of Target Location Frequency [*F*(1,23) = 0.65, p = 0.424, η^2^_p_ = 0.02]. Furthermore, no significant interaction emerged between the two factors [*F*(1,23) = 0.58, *p* = 0.451, η^2^_p_ = 0.02] (Fig. 3).

## Discussion

In the current study, we aimed to assess the combined effects and neural correlates of top-down AC and SL, when both are present. To that end, we manipulated top-down AC via endogenous cueing, and we introduced an imbalance of in-target frequency across locations in the same visual search task. Critically, we implemented the target location imbalance only for neutrally cued trials in order to fully dissociate target location frequency and cue validity in our task. Furthermore, we utilized a neutral rather than an invalid cue as a baseline. Importantly, to be able to compare and unify results regarding the interaction between different AC mechanisms, and to shed light on the functional architecture of visual spatial attention, we used the same visual search task (with some adjustments due to methodological reasons) already implemented and adapted for the study of the integrated effect of other AC signals, namely top-down control via endogenous cueing and bottom-up allocation of attention due to salience (Beffara et al., 2022; Rashal et al., 2022).

### Combined effect of top-down control and SL on behaviour and on N2pc

The behavioural results concerning SL and top-down control confirmed our hypotheses and were in line with the literature, showing an overall effect of both mechanisms of facilitation of target identification following valid, compared to neutral, cues (e.g., Folk et al., 1992; Posner, 1980; Rashal et al., 2022), as well as targets presented in the high- (vs. low-) frequency location (Ferrante et al., 2018; Geng & Behrmann, 2005). Participants could indeed benefit from the available information and prepare for the onset of the array, and then efficiently identify the relevant item (i.e., target). Furthermore, participants’ performance indicated that they had learnt the bias induced by the statistical imbalance of target frequency across locations, which could facilitate target detection in the location where it was more likely to appear. During the debriefing at the end of the experimental session, only four subjects reported having noticed the manipulation. The main results did not significantly change by excluding their data, supporting the idea that people can implicitly extract regularities from their external environment even without explicit instructions (Ferrante et al., 2018; Fiser & Aslin, 2001; Jiang, 2018; Saffran et al., 1996).

Most importantly, in this study we directly examined whether these two AC signals affect attentional deployment in an independent manner when acting together, or whether the effect of one signal interferes with the effect of the other. Our results were more in line with the latter hypothesis, revealing an interaction between the two sources of AC. In particular, at the behavioural level, we observed a gating effect of top-down control over SL, as the effect of SL clearly emerged only in the absence of top-down guidance; responses were faster for targets in the HFTL compared with the LFTL following a neutral but not a valid cue. The prevalence of top-down control could represent an important feature of the functional architecture of visual spatial attention, and it could reflect the ability of voluntary control to actively inhibit (or at least reduce) the contribution of other signals, for example, SL, through a gating mechanism in order to fully and efficiently guide attention to current objectives.

Previous studies, however, are more in line with the idea that top-down control and SL are independent mechanisms and, when active together, the effects of the two are summed-up to bias the competition over attentional resources (Gao & Theeuwes, 2020; Geng & Behrmann, 2005). Gao and Theeuwes (2020) argued that at the level of neural activity, statistical learning creates an implicit landscape where multiple spatial locations have a certain level of activations and inhibitions, and top-down control may then operate to orient the attentional spotlight from one location to another (Gao & Theeuwes, 2020). Their theory seems to be in line with our EEG results. Indeed, even if in our study the final attentional choice seems to be guided by top-down control that prevails over SL in determining performance (e.g., RTs), the EEG data showed that SL is not completely overridden by top-down AC, as an interaction is demonstrated by the N2pc modulation.

That is, in terms of the N2pc, the benefit of cueing the target location was associated with a larger N2pc that was elicited by targets following a valid, compared to a neutral, cue. In addition, this effect interacted with SL, showing an increased N2pc amplitude for validly cued targets (compared with neutrally cued targets), but only when shown at a low-frequency location. This appears to be in line with the priority map theory, whereby the low-frequency target location in general should be associated with less neuronal activation due to the statistical learning mechanism, and thus can benefit from the allocation of top-down attentional resources, as guided by a valid cue. In contrast, when the target appeared at the high-frequency location, any potential benefit from the preceding cue was abolished. This might suggest that the gating effect exerted by top-down AC over SL that we observed on behaviour was not a full gating; on the contrary, at least at some stage along the target selection process the SL was still exerting an effect that could be strong enough to prevent top-down control from emerging (as observed for targets at the high-frequency location in the current study).

Interestingly, the different pattern of N2pc amplitudes observed in the two location-frequency conditions could suggest that a serial search strategy was employed by the participants to find the target (e.g., Woodman & Luck, 2003). That is, the participants may have first searched the high-frequency location before moving to the intermediate- and low-frequency locations. The positive peak observed in the low-frequency condition at the N2pc time-window supports this view (Fig. 3a), considering that the high- and low-frequency locations were positioned at exactly opposite from each other on the screen. Still, this positive peak does not seem to be a fully inverted N2pc, thus, at this point this is only a speculation that could benefit from further investigation.

One could argue that the size of the N2pc was reduced due to the target appearing at the same location repeatedly across consecutive trials, as there was less need for the allocation of attentional resources if attention was already at the correct location. This seems consistent with the cueing effect we observed, since the SL manipulation was present only in the neutrally cued trials. That is, the target was more likely to appear repeatedly at the same location in the neutral cue condition than in the valid cue condition. In contrast, in the valid cue condition there was less chance of repeating target location across trials, as target location was equally probable in that condition. In this vein, the decrease in mean N2pc amplitude in the neutral cue trials is in line with the findings of van Moorselaar and Slagter (2019), who showed a reduction in N2pc amplitude for targets presented at the end of a sequence of targets presented at one location, compared to the targets presented at the beginning of the sequence. However, this alternative explanation cannot account for the SL effect we observed, as it predicts a larger N2pc in the LFTL compared to the HFLT, the latter having more chances of repeating target location in consecutive trials than the former. Evidently, this is the opposite of the pattern we observed here.

One of the main differences between this study and the earlier ones (e.g., Gao & Theeuwes, 2020) is that in our paradigm both top-down control and SL directly determine the search strategy, making it possible to investigate how much SL leaks onto cued trials. In contrast, Gao and Theeuwes (2020) manipulated the frequency of target occurrence across locations, while keeping a location in spatial working memory in order to induce top-down control (Awh & Jonides, 2001; Munneke et al., 2010). Therefore, in their experiment, only SL impacted the search strategy, while the cue determined the second response. Furthermore, in the present study, there was no invalid cue condition and the informative cue predicted the target location with 100% validity. Thus, participants could fully trust the information coming from the top-down control mechanism. Therefore, when these AC mechanisms act together, the interaction between SL and top-down control may depend on the degree of validity of the latter, in turn determining the strength with which it can guide attentional selection even to the point of bypassing the information coming from SL. Together, our findings suggest a close and complex interaction between top-down control and SL, where when one mechanism is acting, and is potentially strong enough to optimize selection, the effect of the other is reduced.

However, we have to note that although the present experiment contained a number of trials that should be sufficient to detect significant effects on the EEG markers of interest with our sample size (Boudewyn et al., 2018), it seems to be limited in its power to capture smaller effects we observe as significant (e.g. Ngiam et al., 2021). Given that a specific target-frequency condition was associated with just one hemisphere depending on the subject group (e.g., HFTL in the right hemisphere), in the current study the N2pc was calculated by collapsing the left and right hemispheres only at a group level (see Wang et al., 2019, for a similar experimental paradigm). This might have made the data vulnerable to increased noise from residual inter-individual variability related to asymmetries in brain activity. This, in turn, might have caused reduced statistical power in some conditions. Therefore, in future studies, larger sample sizes and number of trials might be adopted to increase the robustness of SL and cueing effects studied here.

### Neural activity underlying the preparatory effect of top-down control

In this study, the CNV results reflected processes involved in the preparation of anticipatory attention for the upcoming stimulus and motor preparation needed to respond (Brunia & van Boxtel, 2001; Tecce, 1972). It has been shown that the CNV mean amplitude was modulated by the presentation of a warning stimulus, such as a cue, demonstrating its link to strong attentional engagement (e.g., Rashal et al., 2022; Schevernels et al., 2014; van den Berg et al., 2014). During the CTI, a larger CNV was indeed elicited by a valid (vs. neutral) cue, in accordance with the idea that under top-down control participants could orient their attention in advance toward a certain position. In addition, as a consequence of the preparatory effect exerted by top-down control, we found that the valid cue could also affect the early components of target selection, such as the P1 (e.g., Mangun & Hillyard, 1991). Knowing the upcoming target location allows an early allocation of attentional resources toward a specific region of that display. Indeed, targets following a valid cue produced a general contralateral enhancement of P1 amplitudes. Importantly, we did not find any preparatory advantage due to statistical regularities for the two components; no difference emerged between the CNV elicited by valid cues pointing to the high-frequency location and valid cues pointing to the low-frequency location, and the P1 to the target was not modulated by the target location.

### The priority map is modulated by combined effects of different attention control signals

Most studies of attention control, however, use experimental paradigms that address only one specific AC mechanism at a time, which makes it difficult to understand the contribution of each attentional signal in generating the final attentional choice, and controls how goal-directed behaviour is accomplished by the brain.

Here we used a visual search paradigm previously implemented in its general form in another study that examined the combined effect of different attention-control sources with behaviour and EEG, in an attempt to develop a unified account. Rashal and colleagues (2022) demonstrated that top-down guidance of attention via an endogenous cue diminished the benefit of target salience and the interference from a salient distractor. Similarly, top-down control seems to prevail over the other AC signal, i.e., SL, in this experiment. However, our EEG data showed that SL can block the benefit of a valid cue in the high-frequency location, if in that location the neural activity already reached the highest possible peak due to the probability distribution of target frequency.

As mentioned above, since the present work and the work of Rashal and colleagues (2022) used the same experimental task, it is possible to formulate hypotheses on the functional architecture of visual spatial attention. In particular, together these findings seem to suggest that there is a general dominance of the mechanism underlying top-down allocation of attention, since the effects of both bottom-up capture by salience and SL on performance were diminished, or even absent, following a valid cue. In this scenario, one could argue that the final attentional choice is the result of the activity of only one mechanism, i.e., top-down control, that prevents the influence of all the others. Still, since in our experiment the cue was 100% valid and predicted the target location well in advance, participants could ignore the information coming from the target probability distribution when they already had certain and explicit information about where to find the target. As a consequence, the lack of effect of SL in the valid cue condition was likely not the result of a general gating effect of top-down control, but might simply index that the SL became a condition-dependent mechanism, in this case, following the neutral cue. However, this hypothesis can be excluded given the presence of an interaction between top-down control and SL on the N2pc, which suggests an intervention of SL in assigning different attentional priorities to different locations on the priority map, which can lead to a reduction of the benefit of top-down control during the early stage of target selection.

One critical aspect is that the ability of top-down control to modulate attention and bypass the information of all the other AC mechanisms can depend on its strength and its relevance in the given experimental context. Indeed, a fully reliable informative cue, as the one used in this experiment, can strongly guide attention toward the instructed location without additional information from other AC signals. Contrary to that, when the informative cue is partially predictive (such as is the case where some invalid cue trials occurred), the gating effect of top-down control is weaker since it is needs to also consider information coming from other AC signals, in this case, SL.

## Conclusion

This study seems to indicate an interaction between top-down control and SL, where, when one mechanism is at a play, the influence of the other is reduced or even abolished. In particular, the collected behavioural results suggest that when strong top-down attention control is available, SL could not emerge, as if the information coming from the cue and guiding attention to the indicated spatial location bypasses the information coming from the implicit learning process. Indeed, the fully reliable valid cue allowed participants to pre-allocate their attentional resources to the upcoming target location before the stimuli array onset, thus fully optimizing subsequent target selection. Nevertheless, our EEG results suggest that SL was not totally overridden, at least at some stages of target selection, and in turn being able to reduce the impact of top-down guidance in some cases.

## Footnotes

1 In order to maintain the same experimental protocol as in our previous related work, the cap was placed slightly further to the back than typical, positioning FCz at the Cz site (Rashal et al., 2022). Thus, to test the N2pc and P1 components we report the results from the average between P3/P5 and P4/P6, which are the channel pairs that were closest (~1 cm off) to the PO7/PO8 sites. Similarly, for the CNV the channel used was FCz, which directly corresponds to Cz on the scalp (Verleger et al., 1999; Rashal et al., 2022). For simplicity, we refer to the actual scalp locations, rather than the labels on the cap.

2 To examine whether the SL effect was location-specific or, instead, if it was linked to a hemifield-based representation of space, we repeated the same analysis on the two intermediate locations that were associated with the hemisphere that contained the HTLF and LTLF of each participant. We performed a 2 × 2 rm-ANOVA with Cue (valid, neutral) and Target Location Frequency (intermediate-high, intermediate-low). For accuracy, this analysis showed a significant main effect of Cue [*F*(1, 23) = 10.43, *p* = 0.003, η^2^_p_ = 0.31], but not of Target Location Frequency [*F*(1, 23) = 0.16, *p* = 0.69, η^2^_p_ = 0.006], and no significant interaction between the two factors [*F*(1, 23) = 1.68, *p* = 0.20, η^2^_p_ = 0.06]. However, for RT, significant main effects were found for Cue [*F*(1, 23) = 97.04, *p* < 0.0001, η^2^_p_ = 0.80] and Target Location Frequency [*F*(1, 23) = 8.27, *p* = 0.008, η^2^_p_ = 0.26], but the interaction between these factors was not significant [*F*(1, 23) = 2.21, *p* = 0.15, η^2^_p_ = 0.08]. In contrast to the main analysis, the effect of Target Location Frequency was in the opposite direction, showing shorter RTs in the intermediate-low (vs. intermediate-high) condition. Since in the present study we wanted to assess the top-down control in the presence of a clear SL effect, we did not consider the intermediate frequency locations further.

3 A 2 × 2 rm-ANOVA with Cue (valid, neutral) and Target Location Frequency (high, low) was performed excluding the four subjects who reported having explicitly noticed the target frequency imbalance. For RTs, the analysis confirmed the two main effects [Cue: *F*(1,19) = 115.04, *p* < 0.001, η^2^_p_ = 0.85; Target Location Frequency: *F*(1,19) = 10.92, *p* = 0.003, η^2^_p_ = 0.36] and a significant interaction [*F*(1,19) = 10.15, *p* = 0.004, η^2^_p_ = 0.34]. A post hoc t-test also confirmed the prevalence of top-down AC over SL, which could be seen only in the neutral cue trials (neutral: [*t*(19) = -3.41, *p* = 0.002, Cohen’s *d =* -0.43, -40 sec]; valid: [*t*(19) = -1.57, *p* = 0.13, Cohen’s *d =* -0.08, -7 ms]). However, for accuracy we only found a main effect of cue [Cue: *F*(1,19) = 10.63, *p* = 0.004, η^2^_p_ = 0.35; Target Location Frequency: *F*(1,19) = 1.23, *p* = 0.28, η^2^_p_ = 0.06], and no significant interaction [*F*(1,19) = 0.41, *p* = 0.52, η^2^_p_ = 0.02].
